# Blockchain-Based Data Sharing and Trading Model for the Connected Car

**DOI:** 10.3390/s20113141

**Published:** 2020-06-02

**Authors:** Byeong-Gyu Jeong, Taek-Young Youn, Nam-Su Jho, Sang Uk Shin

**Affiliations:** 1 Department of Information Security, Graduate School, Pukyong National University, Busan 48513, Korea; holine0622@pukyong.ac.kr; 2 Electronics and Telecommunications Research Institute (ETRI), Daejeon 34129, Korea; taekyoung@etri.re.kr (T.-Y.Y.); nsjho@etri.re.kr (N.-S.J.); 3 Department of IT Convergence and Application Engineering, Pukyong National University, Busan 48513, Korea

**Keywords:** blockchain, data marketplace, DO-ABE, ABE, connected car, vehicle data sharing

## Abstract

Currently, “connected cars” are being actively designed over smart cars and autonomous cars, to establish a two-way communication network between the vehicle and all infrastructure. Additionally, because vehicle black boxes are becoming more common, specific processes for secure and efficient data sharing and transaction via vehicle networks must be developed. In this paper, we propose a Blockchain-based vehicle data marketplace platform model, along with a data sharing scheme, using Blockchain-based data-owner-based attribute-based encryption (DO-ABE). The proposed model achieves the basic requirements such as data confidentiality, integrity, and privacy. The proposed system securely and effectively handles large-capacity and privacy-sensitive black box video data by storing the metadata on Blockchain (on-chain) and encrypted raw data on off-chain (external) storage, and adopting consortium Blockchain. Furthermore, the data owners of the proposed model can control their own data by applying the Blockchain-based DO-ABE and owner-defined access control lists.

## 1. Introduction

The fusion of vehicles and information technology has led to the creation of smart cars [1]. Smart cars use a global positioning system (GPS) and geographic information system to determine their current position. A smart car can avoid obstacles in blind spots using the sensors attached to it, and the braking system of a smart car operates automatically when an imminent collision is sensed. In addition, the vehicle infotainment and telematics functions can perform a variety of services, such as social networking service, games, and multimedia, which make the vehicle more than a simple means of transportation. Following the development of smart cars, autonomous cars, which combine smart cars with deep learning and image processing technology, have made it possible to drive without driver intervention [2]. Consecutively, connected cars, an active research and development area facilitated by the fifth-generation (5G) mobile communication technology [3], are being designed for hyper-connection that establishes a two-way communication network between the vehicle and the associated infrastructure [4].

However, some limitations exist that hinder the complete realization of connected cars. Substantial amounts of vehicle data must be stored efficiently and securely, and new intelligent transportation systems and Internet of Everything infrastructures must be established. In addition, vehicle malfunctions caused by hacker attacks can lead to serious accidents; therefore, issues pertaining to both 5G technology and vehicle network security must be addressed. In other words, to realize a connected car, a specific process for secure and efficient data sharing and transaction in a vehicle network must be presented.

According to the trend monitor of the market research company Macromill Embrain, the necessity and installation rate of the vehicle black box are increasing every year [5]. In addition, the utility of the vehicle black box, such as the reduction in the number of unscrupulous drivers and traffic crimes, is also very positive, and concerns about privacy infringement and abuse have been decreasing every year. However, a black box system connected to a connected car can be hacked, and therefore, security mechanisms and solutions tailored to the car network and environment are required. Thus, for a more effective and secure use of a vehicle black box data, a security solution that meets the security and privacy standards must be devised.

Due to the limited storage space, the entire history of the vehicle data are not stored in the connected cars. In addition, the data owners have no choice but to trust a third party for storing the data because they cannot control the outsourced data directly [6]. Furthermore, no vehicle forensic solution is available to resolve vehicle accidents, such as hit and run accidents and vehicle crashes, except for information from external sources such as surveillance cameras and eyewitnesses. The existing vehicle security technology commonly used in connected cars is not well-suited for fast network environments and complex connected car systems. The current vehicle security and privacy protection methods rely on a centralized communication model that identifies, authenticates, and connects all vehicles through a central cloud server [6]. Moreover, most of the current secure communication architectures do not consider user privacy. The centralized model is not scalable because of the large number of connected vehicles, which can lead to bottlenecks and single points of failure in the cloud server, thereby disrupting the entire network [6].

A blockchain-based vehicle data sharing service can resolve the limitations of the existing centralized cloud-based model. To share and trade vehicle data using a Blockchain rather than a centralized model such as the cloud, the following requirements must be met:

- Fairness: Before performing a trade, the commodity and the price must be agreed upon. In our case, the commodity being vehicle data, we can define a condition *V* that must be satisfied by the data. Thus, *V* and price *P* are decided by the data owner *DO* and the data consumer *DC* before trading. We call the trade fair if either DC receives the data satisfying *V* and DO receives *P*, or neither receives any of it. Any entity should be able to pull out of the trade unilaterally, which ensures the timeliness property. An example of *V* would be a hash value check to ascertain the correctness of the data received or access privileges satisfying the access structure. Under the assumption that the hash function *H* is collision-resistant, a malicious party can deceive the agreement over *V* with a negligible probability.
- Transparency, privacy, and security: Vehicle data and vehicle black box video data contain sensitive information; therefore, it is necessary to protect the privacy of such data, and the information that is traded should not be leaked to anyone except DC. Only authorized entities can access the information and only if the trade was successful, which would make the system preserve the privacy. Additionally, the data cannot be modified or altered by malicious attackers. A public log must be maintained to ascertain the ownership of the data. We further require transparency in regarding price and terms of use where, in a trade between the service provider and the DC, the DC should know the original price, and the terms and conditions of the trade between the service provider and the DO.
- Definition of data as evidence of vehicle accidents: Black box data (including video), data from an event data recorder, and basic safety message (BSM) data extracted from on-board diagnostics in a vehicle can prove to be important evidences for vehicle accident investigation [7]. For such data to be effective as evidence, they must meet certain objective qualifications, such as data integrity, legitimacy of the evidence collection process, and authenticity of the evidence extraction system or device.
- Data owner’s control and management of the data: The data owner should be able to claim, control, and manage the data.

In this paper, we propose a Blockchain-based vehicle data marketplace platform model along with a data sharing scheme using Blockchain-based data-owner-based attribute-based encryption (DO-ABE). The proposed model satisfies all the above-mentioned requirements. The proposed system securely and effectively handles large-capacity and privacy-sensitive black box video data by storing the metadata on Blockchain (on-chain) and encrypted raw data on off-chain (external) storage, and adopting consortium Blockchain. In addition, the data owners of the proposed model can control their own data by applying the Blockchain-based DO-ABE and owner-defined access control lists (ACLs).

This paper is organized as follows. In Section 2, we discuss the related works such as connected car, consortium Blockchain, DO-ABE, and off-chain. In Section 3, we propose a Blockchain-based connected car data sharing model. In Section 4, we propose a data sharing scheme using Blockchain based DO-ABE. Finally, in Section 5 and Section 6, respectively, model analysis and conclusions are presented.

## 2. Related Works

### 2.1. Connected Cars

Connected cars aim for a hyper-connection that establishes a two-way communication network between the vehicle and the related infrastructure [4]. They have numerous network structures inside and outside the vehicle. The in-vehicle network connection comprises Ethernet, which is a fast, lightweight, cost-effective, reliable, and scalable technology [8]. A vehicle-compatible gateway is used to interconnect with the vehicle’s external network, which saves the bandwidth and enables edge computing to process the data.

Connections outside the vehicle are divided into connections with other vehicles, cloud storage, mobile devices, and roadway infrastructure and information network [9]. The connection to other cars, i.e., vehicle-to-vehicle (V2V), for safety, V2V applications, and ad-hoc networks operate on an 802.11p & WAVE network. The connection to cloud for content, communication, and safety utilizes cellular, Wi-Fi, and GPS, among others. Mobile devices with application integration, smartphones, and tablets can be connected via USB, Bluetooth, or Wi-Fi. Furthermore, the connection to roadway infrastructure and information network for consumer or commercial information, roadway information, events, warnings, and guidance employs vehicle-to-infrastructure (V2I) and 802.11p & WAVE network.

### 2.2. Consortium Blockchain

The Blockchain is a decentralized record database. It is the underlying technology of Bitcoin, which was developed by Satoshi Nakamoto in 2008 [10]. A data unit called a “transaction” is collected and placed in blocks by the miner node, where timestamp values and hash functions are used to form a chain with the previous block. The Blockchain is also called a public ledger because all transactions and blocks are transparently disclosed to all nodes in the blockchain network. The Blockchain system adopts a peer-to-peer network that operates without a centralized trusted third party, which includes a single point of failure. Furthermore, as a chain-like connected structure, the entire system changes when one block of data are changed. Therefore, the integrity and immutability of the stored data are guaranteed. In its early days, the Blockchain technology was used only for developing crypto currencies. However, with the continuous research on bitcoin and Blockchain, many applications based on the Blockchain have emerged that leverage its decentralized database. Private Blockchain complements problems in the public Blockchain; it allows only selected nodes to access (verify the transaction) the blocks [11].

Blockchains are largely classified into public, private, and consortium Blockchains. Public Blockchains, such as Bitcoin and Ethereum, are Blockchains where anyone can participate as a node. In addition to the transaction details, all actions in the network are shared and can be tracked; however, these are characterized by the disadvantage that the transaction processing speed is slow. Private Blockchain means a closed Blockchain, in which only predetermined organizations or individuals can participate. Except by authorized participants, the transaction details and various actions are not shared and cannot be tracked, and cryptocurrency is not necessary for operation. In a private Blockchain, only the participants approved by the central manager can participate in block creation. Fairness and scalability are complemented by private Blockchains, and direct transactions between institutions can reduce the transaction fees and shorten the transaction times for third parties. Consortium Blockchain is a Blockchain that allows several companies and organizations with the same purpose or value to form a consortium and operate within it [11]. Therefore, consortium Blockchains are more effective than private Blockchains in areas that require consultation by multiple participants. However, for the consortium Blockchain to function effectively, it must have an appropriate governance model and a policy that can be trusted by the blockchain network participants.

### 2.3. DO-ABE

In 2015, Jindan Zhang proposed the DO-ABE to address the shortcomings of the attribute-based encryption (ABE) technique [12]. The existing ABE system requires the existence of a private key generator (PKG) to generate a secret key for the user [13]. In contrast, the DO-ABE utilizes the randomness used for encryption to generate a secret key for the user. Compared with traditional ABEs, DO-ABEs can easily achieve message level-based granular control over the encrypted content. The DO-ABE is divided into key policy DO-ABE (KPDO-ABE) and ciphertext policy DO-ABE (CPDO-ABE). The encryption algorithm distributes the random exponent $s\in Z_{p}$ according to the linear secret sharing scheme (LSSS) access matrix *M*. Private keys are randomly assigned to avoid collision attacks. There are four main functions in the CPDO-ABE (for more details, refer to [12].)

1. Setup(*U*): The setup algorithm takes the number of attributes, *U*, in the system as input. It then selects a group *G* of the prime order *p*, a generator *g*, and *U* random group elements $h_{1},h_{2},...,h_{U}\in G$ associated with the *U* attributes in the system. The public parameter is $\left(g,h_{1},h_{2},...,h_{U}\right)$.
2. KeyGen(*MSK*, *S*): The encryption entity (data owner) selects a random index $\beta ,\alpha \in Z_{p}$ and computes the public key $PK=({e(g,g)}^{\beta },g^{\alpha })$. The owner entity sets $MSK=g^{\beta }$ as the master secret key and takes it with a set of attributes *S* as input. Then, the user secret key SK is generated using the following equation:
  (1) $$K=g^{\beta }g^{\alpha t},L=g^{t},\forall x\in S,K_{x}=h_{x}^{t},$$
  where *t* is a random element in $Z_{p}$.
3. Encrypt(*PK*, *ST*, *m*): The encryption algorithm takes the public key PK and the message *m* to encrypt as the input. In addition, the LSSS access structure ST=(AM,p) is received as input. Now, AM is the l×n matrix. The algorithm selects a random vector $\bar{v}=\left(s,y_{1},y_{2},...,y_{n}\right)\in Z_{p}^{n}$ and a random $r_{1},r_{2},...,r_{l}\in Z_{p}$. The ciphertext is published in CT with ST as follows:
  (2) $$C=m\cdot e{\left(g,g\right)}^{\beta s},C^{{}^{\prime }}=g^{s},$$
4. Decrypt(CT, SK): The decryption algorithm takes the ciphertext CT for the access structure ST and the secret key SK for the set *S* as input. The decryption algorithm first computes the following equation:
  (3) $$\frac{e(C^{{}^{\prime }},K)}{\prod_{i\in I}({\left(e\left(C_{i},L\right)e\left(D_{i},K_{p(i)}\right)\right)}^{\omega_{i}}}=\frac{e{\left(g\cdot g\right)}^{\beta s}e{\left(g,g\right)}^{\alpha st}}{\prod_{i\in I}e{\left(g,g\right)}^{t\alpha \omega_{i}\lambda_{i}}}=e{\left(g,g\right)}^{\beta s},$$
  where $\omega_{i}\in Z_{p}$ is a set of constants such that if $\lambda_{i}$ are valid shares of any secret *s* according to AM, then $\sum_{i\in I}\omega_{i}\lambda_{i}=s$. The decryption algorithm then divides this value by *C* to obtain the message *m*.

The data consumers share the same attribute values; however, the data owner can choose these arbitrarily, resulting in different data. Thus, the DO-ABE is more flexible in accessing control than conventional ABE. Due to this flexibility, DO-ABE is an encryption technique that is suitable for applications such as cloud storage.

### 2.4. Off-Chain Technology

Blockchain ensures high availability of the applied system and immutability of the stored data. However, the properties of decentralization, scalability, and security cannot be satisfied simultaneously; this is known as the Blockchain trilemma [14]. In particular, the problem of scalability has implications in Blockchain applications in finance, distribution, and healthcare, which deal with vast and diverse types of data.

Off-chain technology denotes the ways in which some of the work performed on the existing main Blockchain can be performed outside the Blockchain [15]. Off-chain technology can partially solve the Blockchain scalability problem. The reference values of the stored data are stored on-chain and the encrypted original data are stored off-chain. Therefore, it achieves privacy features that can prevent data from being disclosed directly. Moreover, it can improve the speed of transaction processing by reading the target value from the on-chain to perform the state change transaction and reflecting only the value of the calculated result on the on-chain. Some methods are available for performing off-chain transactions by forming channels between two parties or using separate sidechains. Similar to that in the main Blockchain, it can use public and private smart contracts to synchronize the on-chain and off-chain states and ensure data privacy. A general function or a function that takes an open value as input is distributed in a public contract. When speed is important or high computational costs are required, or when a secret input value must be included, a private contract is distributed. Public contracts are used by all nodes on the chain, whereas private contracts are used only by off-chain nodes.

**Figure 1:**
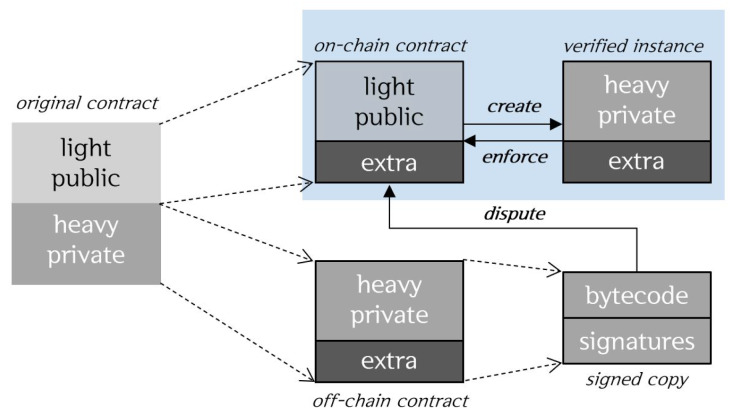
On/off-chain smart contracts [16].

Li et al. proposed the design of on/off-chain smart contracts [16]. They divided the functions performed by smart contracts into two types and executed them on-chain and off-chain each, to establish a hybrid smart contract system with privacy protection. Functions of a smart contract are classified into the two categories: (1) heavy/private functions involving high-cost computation or sensitive information and (2) light/public functions involving none of these features. In addition, the light/public functions and heavy/private functions are depolyed to on-chain contract and off-chain contract, respectively. On-chain contracts are deployed to be publicly executed by all the miners while off-chain contracts are privately executed only by a small number of interested participants. Figure 1 shows the classification of contracts. Adopting such a hybrid smart contract can speed up consensus and not reveal sensitive information to the public Blockchain.

## 3. Blockchain-Based Connected Car Data Sharing Model

This section introduces a model for sharing and trading several types of vehicle data (including vehicle black box video data) generated by connected cars. First, the assumptions and the trust model are presented, and the types and characteristics of the system participants are discussed. Then, the reasons for adopting a consortium-type Blockchain, consensus mechanism, structure of blocks and transactions, smart contracts, and private contracts are explained.

### 3.1. Assumption and Trust Model

We consider a Blockchain model comprising data owners (producers) and data consumers. The data owners primarily store their original data on a cloud storage or an InterPlanetary File System(IPFS) that can harness and process large amounts of data. The metadata of the original data are stored in the Blockchain. Our model adopts the consortium-type Blockchain that may require collaboration among the competing participants [17]. With different objectives and priorities, none of the participating organizations wishes to relinquish control of the consortium. For the consortium Blockchain to function effectively, it is necessary to have a governance model. For example, one alternative could be that a legally separate agency or Blockchain association oversees the consortium. In addition, there is a need for policies for consortium management, technical infrastructure, participant compliance, and arbitration in disputes that can be trusted by the participants in the consortium Blockchain network. However, this proposal model does not cover the subjects of governance and policy. It is also assumed that the institutions participating in the consortium are sufficiently honest to reach a correct consensus and that the Blockchain system that is achieved by these participants is stable. The consortium-type Blockchain allows only authorized nodes to participate. To this end, a trusted authority exists to authorize the participating nodes. Therefore, in the proposed model, it is assumed that a trusted authority exists and the participating entities register with the trusted authority and receive information such as a unique ID. Over Blockchains, all members can be either a data consumer, or a data producer, or both. Both the owners and the consumers hold unique membership identities over the Blockchain network. Identity establishment relies on the existing Membership Service Provider (MSP) or Certificate Authority (CA) that is often a part of Blockchain implementation. MSP or CA generates and manages identities, keys (public and private), and addresses for all nodes in the network.

Connected cars still require intensive research regarding security. In fact, in July 2015, two hackers hacked a Jeep Cherokee and manipulated its engines, brakes, wiper controls, and GPS [18]. Multifaceted security technologies are required for modern vehicles to be networked. Research is being conducted in various fields such as network security, onboard communication security, security for mobile access, and trusted authentication methods [19]. Professional hardware and software are required, along with existing solutions, to meet the requirements of the international standard ISO 26262 titled “Road vehicles—Functional safety”. The proposed model assumes that the network inside and outside the connected car is secure, and that the sensors and controllers that generate the data are reliable, unmodified, and not extorted by hackers.

With the evolution of vehicle black boxes, network-enabled Wi-Fi black boxes are emerging. Furthermore, the following assumptions are made: (a) the manufacturers of vehicle black boxes are trusted, and the vehicle black box video data are reliable and remain unmodified; (b) the wireless networks of the devices (including the vehicle black box) mounted on the connected car are secure; (c) the on-device data storage is secure against malicious attacks; and (d) the reliability and security of the device itself is ensured by techniques such as Trusted Platform Module and remote attestation.

In this paper, we assume the following threat model: First, the Blockchain that ensures immutability and verifiability is trusted by all participating entities. There is no trust between the data owner and the consumer, but the two parties use the core properties of the Blockchain for security. Before sharing the data, the data consumer establishes a trading transaction with the data owner according to the data offer previously suggested by the owner, and then the transaction verified by both parties is recorded on the Blockchain. Next, it is assumed that the consumer may be a malicious attacker who attempts to illegally access data or modify the ACL for unauthorized access to the data. The ACL is managed by transactions issued by the data owner, which are recorded on the Blockchain, so an attacker must compromise the security of the Blockchain in order to alter the ACL. For example, in the case of a Bitcoin blockchain, attackers must control more than half of the voting power of the blockchain network to succeed in this attack.

### 3.2. System Model

**Figure 2:**
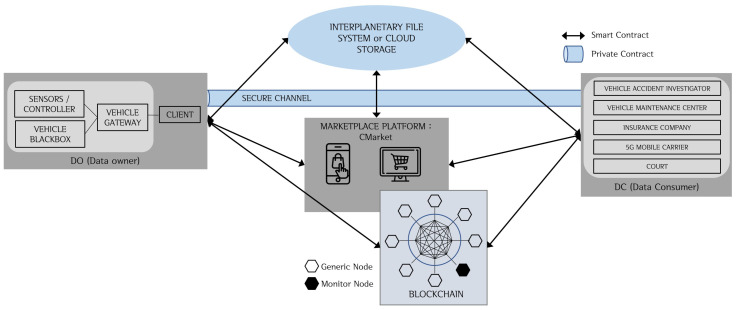
System model.

The proposed system is shown in Figure 2. It consists of a data owner (DO), data consumer (DC), blockchain network, marketplace platform, and external storage such as IPFS or cloud storage.

#### 3.2.1. System Participants

A data owner, data consumer, and service provider are the system participants. The data owner provides the vehicle data and vehicle black box video data, the data consumer requires the data, and the service providers help connect and establish trade between data owners and data consumers.

The data owner is the owner of the data collected by the black box or the connected car. The data owner can provide the collected data to the blockchain network and the marketplace platform.

The data consumers are entities that require the vehicle black box data, and include vehicle accident investigators, vehicle maintenance companies, insurance companies, mobile carriers, and judicial authorities. They form a consortium and participate in the Blockchain system. They can find the data they need on the marketplace platform, and access them by providing appropriate means of access, such as warrants or certificates. When a transaction is made, the data consumer is given the location and the key to access the original data. Car accident investigators, car maintenance companies, insurance companies, and 5G mobile carriers are general nodes that agree and verify blocks and propose blocks to the Blockchain system. Courts are monitor nodes that do not directly participate in the consensus process of the Blockchain system but are allowed to participate in post-incident disputes through a replica of the shared ledger [6].

The service provider is a Blockchain-based marketplace platform. The marketplace platform is described in detail in the following section.

#### 3.2.2. Marketplace Platform

Marketplace platform (CMarket), a service provider of the Blockchain-based connected car data-sharing model, is a decentralized application (DApp) implemented as a web or application that data owners register data and helps data consumers retrieve data. CMarket is a service provider that mediates the transactions between the data consumer and the data owner; put differently, CMarket functions as a broker or mediator. It manages the account information on the consumer’s behalf, helping make payments and transactions quickly and easily. After the data consumer pays via an appropriate payment method, the data owner communicates the location and the key of the encrypted original data through a secure channel. CMarket supports a secure channel between the data owners and the data consumers for off-chain transactions. Similar to most DApps, CMarket is released as open source. Anyone can verify the CMarket because its source code is open. The implementation of CMarket follows the standard development protocol. It is assumed that CMarket is a semi-trust (honest-but-curious) entity.

**Figure 3:**
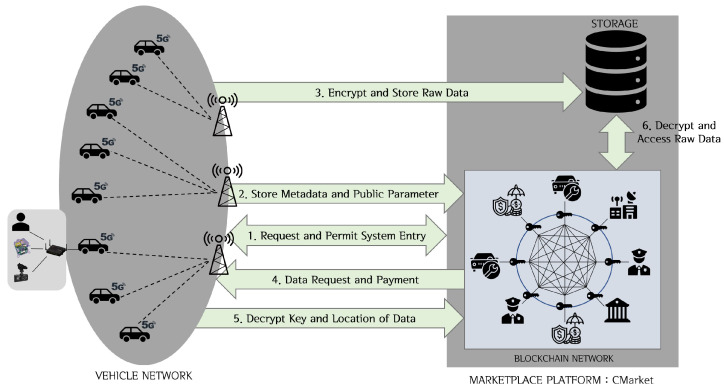
Marketplace Platform: CMarket and Data Flow.

Figure 3 shows the structure and transaction flow of CMarket. First, the connected car’s vehicle gateway collects and preprocesses the data generated by the vehicle and the black box. These processed data are transferred from the vehicle gateway via CMarket to the blockchain network and external storage via 5G wireless communications. CMarket receives the processed vehicle data and black box video data and then sorts the data by the relevant characteristics for outputing them on a screen for a more efficient data search or manipulation. Data consumers can use CMarket to find the data they require and obtain them from the owner.

For development and implementation of CMarket, Hyperledger Fabric [20], a representative platform for private Blockchains and consortium Blockchains, can be used.

#### 3.2.3. Type of the Adopted Blockchain and Consensus Mechanism

The main transaction data of the proposed model comprise a vehicle black box video containing large amounts of sensitive information. Therefore, it is necessary to authenticate, verify, and limit the participating entities. Accordingly, the consortium Blockchain is adopted [11]. In this model, consortiums are typically formed by the data consumers, including car accident investigators, insurance companies, car maintenance companies, and mobile carriers. The data owner, the owner of the vehicle black box, or the vehicle gateway participates as a client in the Blockchain system. Car accident investigators, car maintenance companies, insurance companies, and mobile carriers are general nodes that agree, verify, and propose blocks to the Blockchain system. The judicial authorities do not participate in the verification and consensus process, but participate in the system as a simple monitor node that maintains a copy of the shared ledger to resolve disputes at trial.

Considerations for setting the consensus algorithm of the model are as follows:

1. It must be suitable for the consortium Blockchain.
2. Transactions per second should be sufficiently fast to handle the large amounts of vehicle data.
3. Mining compensation may not be required.
4. It must be suitable for Internet of things (IoT) environment.
5. An algorithm in which the Blockchain system nodes (excluding monitor nodes) can participate in the consensus is necessary.
6. An algorithm is required to select a leader from among the nodes that can participate in the consensus.

For a node to participate in the consortium Blockchain system, the trusted authority’s approval is required. The registration of the participating node relies on the trusted authority assumed in the consortium Blockchain. For creating a block, consensus nodes are selected by the trusted authority, which record the block on the Blockchain by the adopted consensus mechanism. The general consensus process in the consortium Blockchain is as follows. A consensus nodes is randomly designated as the leader. The leader node is maintained only for a certain amount of time hard coded into the system; a new node is randomly elected after the time elapses. The fixed retention time prevents the leaders’ malicious behavior. The leader nodes verify and confirm new nodes that request participation in the Blockchain system, and grant Blockchain participation approval. Nodes requesting participation can participate in the Blockchain system with the approval of the leaders. Additionally, leader nodes perform a consensus, verification, and provide suggestions for composing transactions consisting of the metadata of the vehicle data, vehicle black box video data, and hash values, among athers, into blocks of the Blockchain.

The method of reaching consensus among the randomly designated leader nodes of each institution may be a consensus algorithm such as the Practical Byzantine Fault Tolerance (PBFT) [21]. The PBFT consensus algorithm is a mathematically proven algorithm in which, when there are *f* faulty nodes in an asynchronous network, the consensus made in the network is reliable if the total number of nodes is 3f+1 or higher. In other words, the PBFT consensus algorithm guarantees the reliability of the consensus made within the network even if there are some malicious nodes in the network.

Currently, the research on consensus algorithms suitable for IoT environments such as [22] and [23] or those suitable for consortium Blockchains such as [11,24] is in progress. However, it is difficult to apply this consensus algorithm to the proposed model. Therefore, the research on the consensus algorithm suitable for the proposed model is another important individual research topic that must be studied in detail. In this paper, it is assumed that a consensus algorithm such as PBFT is used.

#### 3.2.4. Block and Transaction Structure

**Figure 4:**
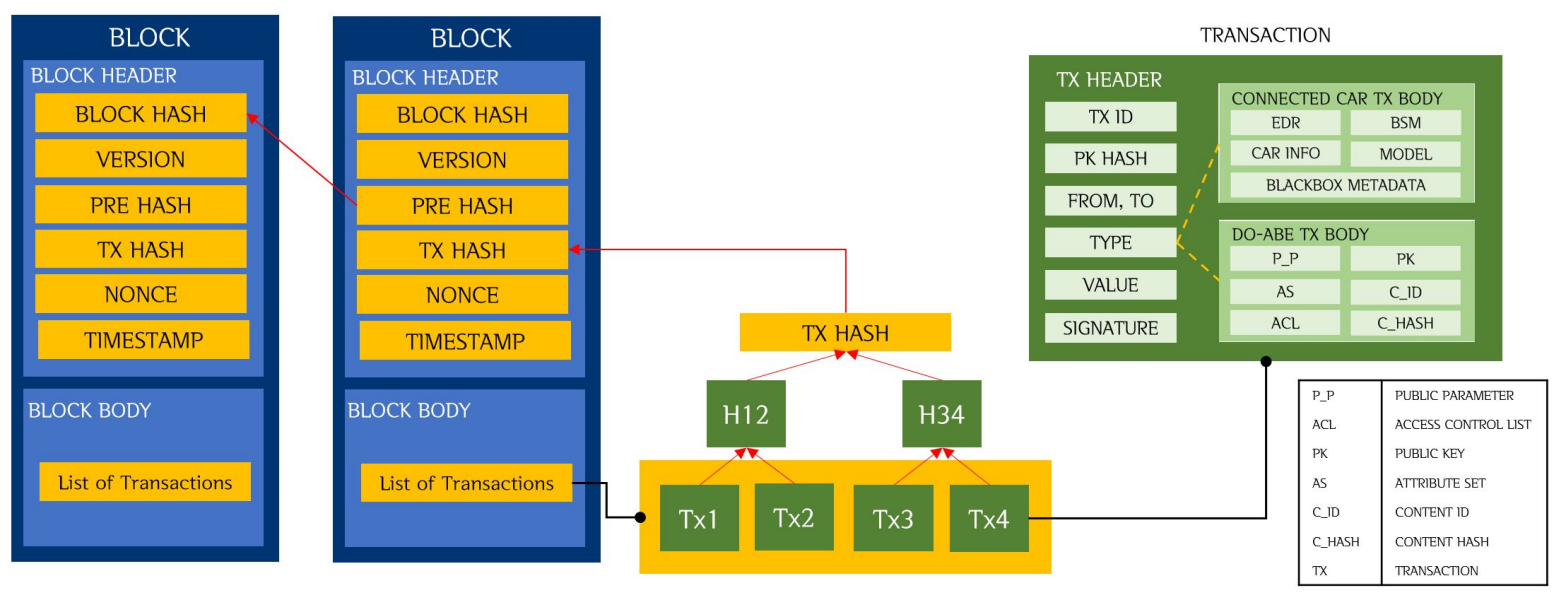
Block and transaction structure.

The data stored in the Blockchain are generated in units of transactions. Figure 4 shows the block and transaction structure of the proposed model.

The transaction is divided into a transaction header and a transaction body. The header part of the transaction contains general items such as the transaction identifier (ID), amount to be sent, digital signature, recipient’s public key hash value, and type [25]. The transaction body contains vehicle-related data, black box video data, data for DO-ABE, or data trading related information. The type in the transaction header identifies the information contained in the transaction; thus, transactions are divided into connected-car transaction, DO-ABE transaction, and trading transaction.

Because Blockchain is irreversible, data can only be added, not modified or deleted [11]. The smaller the size of the data stored in the Blockchain, the better the block scalability, the higher the transmission success rate, and the lower the transmission delay [14]. Therefore, only the metadata related to vehicle data and black box video data are stored in the Blockchain, and the original data are stored separately in external storage. The connected-car transaction includes the vehicle information generated by the black box, BSM, DSRC, and vehicle type. The data related to the black box video include video time, travel path including the departure and arrival locations, size of the video, and description. The DO-ABE transaction additionally includes DO-ABE related data. The transaction data related to DO-ABE are described in Section 4.

These transactions are collected into a block, which consists of a header part and a body part. The body part includes the list of the above-mentioned transactions; the header part includes the hash value of the transaction list, hash of the previous block, timestamp, and  version.

Table 1 lists the size of the data included in a block and a transaction. The size of each data sample is a general number and an estimate calculated by the data type. Assuming that there are 10 consumer node lists (number of attributes *U*) defined by the owner, when calculated as the maximum value of each data, the block header size is 106 bytes and the transaction header size is 166 bytes. In addition, the size of the connected car-related transaction body is 512 bytes; and that of the DO-ABE-related transaction body is 584 bytes. The size of a block with four transactions is approximately 3.6 KB; this size can contain about 1460 transactions when the block size is set to 1 MB.

#### 3.2.5. Smart Contracts and Private Contracts

Smart contracts are programs that are registered and run on the Blockchain, forcing the execution of certain functions [26]. In this model, transactions that are not large and need to be enforced on the contract are processed by smart contracts. This includes registering, redefining, and verifying the ACLs defined by the data owner, constructing new data sets, and processes necessary for data trading.

The Algorithm  1, 2 and 3 represented in pseudocode, describe smart contracts. CreateData in Algorithm 1 is a function by which the data owner creates and stores data, which can only be executed by the owner. It obtains the data owner’s ID and the data as a parameter. First, it checks whether the data owner is a node participating in the Blockchain system. If found to not be an authorized participating node, this function is stopped; if found to be an authorized participating node, it stores the data on the Blockchain and external storage. GetData in Algorithm 2 is a payable function that accesses and obtains the data that a consumer requires. It obtains the data consumer’s ID and an access privilege such as a certificate or a warrant as a parameter. First, the data consumer’s ID and the access privilege are verified. If the consumer is unauthorized or if the access privilege is not appropriate, access is denied. If the verification of access is successful, the consumer pays the owner a data value. After confirming the transaction, the data owner communicates the storage location of the original data to the consumer. The consumer obtains the encrypted original data and can decrypt it with the data key obtained through the appropriate access attributes. HandlingACLs in Algorithm 3 is functions related to ACLs defined by the data owner; thus, this function can be executed only by the owner. It takes the ID of the data consumer as a parameter. First, check if the ID of the data consumer is included in the existing ACL; if present, the data key is computed and returned. If not, the owner checks the latest payments made by the consumer from the transaction. If the transaction is valid, the data owner adds the consumer’s ID to the ACL and uploads the latest ACL on the Blockchain.
**Algorithm 1:** CreateData**Require:** DO’s ID, Data  onlyOwner  **if** Validation(DO’s ID) **then**    MSK,PK=KeyGen()    StoreOnChain(PK)    $EF=SymEnc(F,sk_{F})$    $CT=Encrypt(PK,ST,sk_{F})$    metadata of Vehicle data = timestamp, location    metadata of DO-ABE data = UID,Fid,F,H(EF)    StoreOnChain(metadata of Vehicle data)    StoreOnChain(metadata of DO-ABE data)    StoreOffChain(Vehicle data & (Fid,EF,CT))  **else**    REJECT Creating  **end if**
**Algorithm 2:** GetData**Require:** DC’s ID, Proof of Access  payable  **if** Validation(DC’s ID, Proof of Access) **then**    PaytoOwner(data value, DO’s address)    SK,H(F) = GetfromOwner(set of attributes *S*)    Fid,EF,CT = GetfromOffChain()    $sk_{F}=Decrypt\left(CT,SK\right)$    $F=SymDec(EF,sk_{F})$  **else**    REJECT Getting  **end if**

**Algorithm 3:** HandlingACLs**Require:** DC’s ID  onlyOwner  **if** CheckACL(DC’s ID) **then**    Compute(data key)    **return** (data key, location)  **else if** Check(transaction) **then**    AddtoACL(DC’s ID)    StoreOnChain(ACL)    Compute(data key)    **return** (data key, location)  **end if**


In the proposed model, the original data and the metadata are stored separately to overcome the limitations of Blockchain. The metadata of the vehicle data and the vehicle black box video data (including the hash value of the original data) are stored in the Blockchain, and the original data are stored in an external storage such as a cloud storage platform or IPFS [27]. The data consumers can verify the integrity of the data by comparing the hash value of the original data with the hash value of the data stored in the Blockchain. These processes of registering and accessing the data on external storage, transferring data that must be protected for data confidentiality, or requiring enormous computations such as DO-ABE encryption, are implemented as private (off-chain) contracts on a secure communication channel, such as secure sockets layer [28]. The process of registering the data in an external storage is established through a secure communication channel between the owner and the external storage. The data owner extracts the metadata and the hash value of the data, transmits this information to the Blockchain, and then transmits the original data to the external storage. For this purpose, the encryption process must be performed first. The data owner inputs the symmetric key $sk_{F}$ as a parameter; and creates an encrypted file EF with a predefined symmetric key encryption such as AES. In this process, original data and meta data are stored separately for data confidentiality. The original data with large volume is encrypted and stored in off-chain (external) storage, and the metadata are processed as a transaction and maintained in on-chain storage (Blockchain). Unlike the registration process, the process of accessing the data in the external storage is performed through two communication channels between the data consumer and the external storage, and the data consumer and the data owner. First, the data consumer proves to the data owner that they are a legitimate user through the access property set *S*. The data owner verifies the data consumer, and, if found to be a legitimate user, transmits the secret key SK and the address of the external storage where the original data are stored. At this time, the secret key SK is calculated through the KeyGen function predefined in the off-chain private contract. This process requires a separate communication channel between the parties on the off-chain because the secret key SK, which should not be disclosed to third parties, is exchanged. Data consumers who received the necessary parameters for obtaining the original data from the owner can access the external storage, and, therefore, download the encrypted original data. Data consumers who obtained the encrypted data can obtain the original data through a predefined DO-ABE decryption function on the off-chain. When the trading between the data owner and the data consumer is successfully completed with the off-chain contract, the transaction for the processing result is published on the Blockchain. The overall data sharing process, including the on-chain and off-chain processes, is detailed in Section 4.

## 4. Data Sharing Scheme Using Blockchain Based DO-ABE

The data owners merely provide data to the Blockchain system as a client and do not participate in the consensus, verification, and proposal process of the block. This violates the public Blockchain concept of eliminating centralization and managing all the data recorded in the ledger. The proposed model applies a consortium-type Blockchain for the feasibility and efficiency of the system as mentioned earlier. In this model, the data owners use a Blockchain-based DO-ABE to assert and control their own data.

Unlike the conventional ABE, which requires the presence of a PKG to generate a secret key for the user, the DO-ABE utilizes the randomness used for encryption to directly generate the secret key for the user [12]. However, because DO-ABE is an encryption technique suitable for applications such as cloud storage, it must be reconfigured for Blockchain. It separates the function of cloud storage, which acts as storage, into Blockchain and external storage. From the data related to DO-ABE, the public parameters, a set of all attributes, a public key, an identifier of content, and a hash value of the encrypted content are stored in a Blockchain. The identifier of the content, encrypted content, and ciphertext of the symmetric key used for the encrypted content are stored in an external storage.

**Figure 5:**
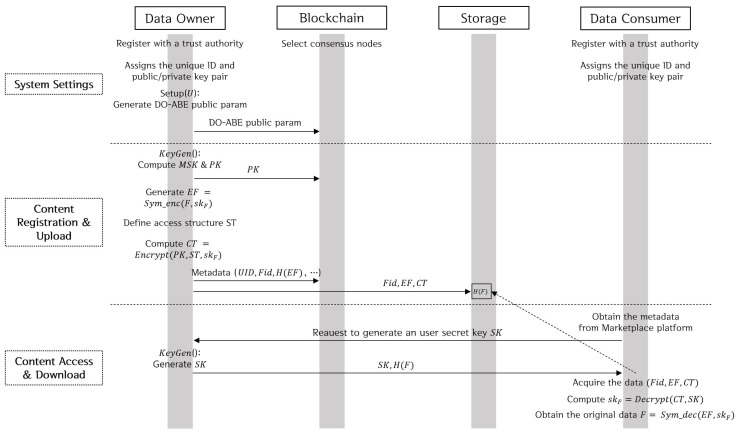
Data Sharing and Trading Process using Blockchain-based DO-ABE.

Figure 5 shows the data sharing and trading process using Blockchain-based DO-ABE. Data owners define the unique identifiers of consumers who complete the trading through smart contracts in an ACL, and then publish it as the Blockchain transactions. The data consumer can access and process the data only if the ACL allows it by the smart contract. These processes are as follows:

1. **System Settings**: The proposed model adopts the consortium-type Blockchain in which a trusted authority exists. Therefore, all entities must be registered with the trusted authority before they participate in the system; and this process assigns a unique ID and a public/private key pair to each entity. In addition, the consensus nodes participating in the Blockchain are selected based on the adopted consensus mechanism. Each data owner generates DO-ABE public parameters to control their data and registers these parameters on the Blockchain through a DO-ABE transaction. For this purpose, the public parameter $\left(g,h_{1},h_{2},...,h_{U}\right)$ is generated through the Setup function described in Section 2.3, and is then published on the Blockchain.
2. **Content Registration and Upload**: First, the data owner computes a master secret key $MSK=g^{\beta }$ and a public key $PK=({e(g,g)}^{\beta },g^{\alpha })$ using the KeyGen function. The generated public key PK is recorded on the Blockchain through a DO-ABE transaction. The ciphertext EF is generated by encrypting the original vehicle data *F* with a randomly generated symmetric key $sk_{F}$ (EF=*Sym-enc*$\left(F,sk_{F}\right)$). After defining the appropriate access structure ST, the ciphertext CT is computed by encrypting $sk_{F}$ using the Encrypt function in Section 2.3 ($CT=Encrypt(PK,ST,sk_{F})$). The metadata including the owner ID UID, identifier Fid of the vehicle data *F*, and hash value H(EF) of the encrypted content are published on the Blockchain through a connected car transaction. The metadata may include timestamp information and location information such as GPS. (Fid, EF, CT) are stored in an external (off-chain) storage. At this time, H(F) may be used as the storage location information.
3. **Content Access and Download**: The consumer obtains the corresponding metadata by searching for the desired content through the marketplace platform using the location information and time information. The consumer with the set of access attributes *S* requests with the data owner of the desired data through the off-chain private contract to generate a user secret key SK for accessing the data. The data owner creates the consumer’s attribute secret key SK ($K=g^{\beta }g^{\alpha t},L=g^{t},\forall x\in S,K_{x}=h_{x}^{t}$) through the KeyGen function and then securely delivers SK and the storage location H(F) to the consumer through the off-chain private contract. The consumer obtains the black box data (Fid,EF,CT) stored in the storage location H(F), and then calculates ${sk}_{F}=Decrypt\left(CT,SK\right)$. The consumer can obtain the original data *F* by decrypting EF with the symmetric key ${sk}_{F}$. When this trading transaction is successfully completed, it is recorded on the Blockchain. Here, the payment process for data purchase can be performed through off-chain techniques such as commit-chain and state-channel [29].

## 5. Analysis and Discussion

Various Blockchain-based service models related to vehicle data have been proposed [6,7,30]. Cebe et al. proposed a lightweight privacy-aware Blockchain composed by all relevant parties, such as drivers, maintenance centers, automobile manufacturers and law enforcement agencies, without requiring a trusted third party [7]. Special forensic daemons are used to provide evidence of vehicle data. It also satisfies privacy through integrated member management and pseudonym certificates. To satisfy the anonymity of the client, the pseudonym identification information of the vehicular public-key infrastructure (VPKI) model proposed in IEEE 1609.2 is used as a token; it uses a lightweight ledger to exchange data safely and efficiently between parties. Javed et al. proposed a Blockchain-based secure data sharing mechanism for vehicle networks [30]. The use of IPFS resolves the problem of centralized storage. Furthermore, Intelligent Vehicle Trust Point was introduced to maintain the vehicle’s trustworthiness and ensure fair payment for a given service. It protects user privacy using an encryption ID. In addition, the communication between the vehicles is encrypted by symmetric key encryption, thereby protecting the privacy of the data. It adopts a permissionless Blockchain to achieve transparency of data and protect data through the distributed storage method of IPFS. Dorri et al. proposed a new vehicle security architecture based on Blockchain [6]. The user’s privacy is protected using a changeable public key. In addition, Overlay Block Managers is introduced to provide cluster members with access control on the transmitted transactions. By adopting an overlay network and a light scalable Blockchain, a Blockchain instantiation optimized for IoT environments is proposed. All transactions in the network are encrypted using an asymmetric encryption technique, and each node is authenticated using a public key. To protect user privacy, an in-vehicle storage is present in each vehicle for storing sensitive data. This storage also provides a high level of privacy by changing the public key for each transaction. Data owners can further control the exchanged data by defining the data to be provided to third parties and the data to be stored in the in-vehicle storage device. However, the existing vehicle data-related Blockchain models do not consider the data owner’s data control or management and the fairness in data transactions. Unlike the existing service model, the proposed model presents a method and a solution for processing not only the data generated in the vehicle but also large and privacy-sensitive data, such as a video in the vehicle black box. The proposed system securely and effectively handles large-capacity and privacy-sensitive black box video data by storing the metadata on Blockchain (on-chain); and the encrypted raw data on off-chain (external) storage; and adopting a consortium-Blockchain. Furthermore, the data owners of the proposed model can control their own data by applying data owner-defined ACL and DO-ABE.

The proposed model addresses the shortcomings of the existing models by satisfying the following basic requirements:

- Fairness: Data can be encrypted while defining specific users and data through attribute values that must be satisfied with the data. The CMarket proposed in this paper, as a service provider, helps transactions between data owners and data consumers. These service providers mediate whether the data consumer has received data that satisfy the attribute value or if the data owner has received the price. Strong fairness is guaranteed under the assumption that the data owner and the data consumer are honest.
- Transparency, Privacy, and Security: Large vehicle data and black box video data are stored separately in an external storage, and the public parameters for generating keys and relatively small metadata are published in the Blockchain. For data privacy, raw data are encrypted with a symmetric key and stored in an external storage. Furthermore, the symmetric key is encrypted and stored using the DO-ABE scheme. Thus, only the entities with appropriate privileges granted by the owner can access the original data. To establish trust between trading parties, we adopt a consortium Blockchain in which only nodes that are already authorized participate. For transparency, all transactions such as data registration, data access, and data trading are recorded in the Blockchain. Thus, by looking into the Blockchain, any party can ascertain the ownership of the data and the history of access to the data. In addition, data integrity is ensured because of the use of Blockchain. Because the hash value of the original data are stored in the Blockchain, the data consumer can verify the integrity of the original data.
- Definition of data as evidence of vehicle accidents: The data retrieved from the proposed model are of significant value as legal evidence. The integrity and traceability required to be admitted as evidence are realized because of the use of Blockchain. Objective qualifications as legal evidence, such as the legality of the evidence collection process and the credibility of the evidence extraction system, can also be satisfied.
- Data owner’s data control and management: To claim data ownership and control data, it uses ACLs that are defined by the data owner and an attribute-based encryption based on the data owner. The data owners generate policies (some attributes set) for their own data, and only consumers satisfying the policies can access the data. In addition, the owner can grant or revoke access by modifying the policy.

Table 2 comparatively analyzes the existing models and the proposed model.

Furthermore, the benefits of the various data consumers are as follows. Vehicle accident investigators can find the data they require through CMarket without having to search for evidence or witnesses at the time and place of the accident and use integrity-assured data as evidence. Features such as transparency, traceability, and integrity of the Blockchain validate the data as evidence [7]. This significantly reduces the time required for investigation because it allows for faster and more accurate analysis of the cause of the accident. Insurance companies and vehicle maintenance centers can use the vehicle data from black box and BMS recorded on the Blockchain for insurance purposes and remote diagnosis, respectively. Mobile carriers can accurately measure the cost of communication and provide high quality customized services based on the data. Judicial authorities can easily verify the integrity of the evidence presented by a vehicle accident investigator and accurately determine the responsibility for a crime or an autonomous vehicle accident from a vehicle black box video.

The property and security of the proposed system and the applied ABE scheme are discussed in more detail. The existing ABE schemes require a PKG to generate a secret key for the user instead of eliminating the public key infrastructure for complex certificate management and public-key issues. However, although ABE can achieve flexible control over the content that can decrypt the ciphertext, it cannot achieve granular message-based control [13]. Jindan Zhang proposed DO-ABE [12], a cloud-storage solution that generates a secret key for users by utilizing the randomness used for encryption. In this paper, we redefined the existing cloud-storage DO-ABE as a Blockchain-based model. In the existing Blockchain data access control scheme based on ABE such as [31], attribute authorities control the distribution of attributes. Thus, data owners have no choice but to rely on attribute authorities to control their data. This is a structure in which one node of the authorities creates a block and processes the transaction with the attribute authorities acting as key distribution centers generating the attribute request transaction. However, the proposed model addresses the shortcomings of the existing models in terms of data ownership because the data owner does not rely on a central trust entity to control the access to their own data. Therefore, the proposed model provides data sharing and transactions more effectively and securely through a Blockchain based DO-ABE than the existing services.

The security proof for Blockchain based DO-ABE is almost the same as that in [12] because our scheme shares the same structure as that in [12]. Thus, the security proof can be directly applied to our scheme (here we omit the concrete proof; interested readers can refer to [12]). Only the authorized (satisfied attribute policy) entities can access the data and obtain the corresponding decryption key. The data owner generates policies (some attributes set); and only consumers satisfying thees policies can access the data. In addition, the owner can grant or revoke access by modifying the policy.

In the proposed scheme, the confidentiality of the data stored on the off-chain storage can be trivially guaranteed against entities who have no attributes that satisfy the access policy. If the set of attributes of an entity cannot satisfy the access policy, one cannot recover the data encryption key and therefore the original content cannot be accessed. The colluding entities who do not have enough attributes to access the data alone may combine their attribute secret keys to decrypt the data encryption key together. To obtain the data encryption key, they must recover $e{\left(g,g\right)}^{\beta s}$ with their attribute secret keys. However, an attribute secret key is randomized with the personalized values *t* generated by the data owner, which is uniquely related to each entity. Therefore, the desired data encryption key cannot be decrypted by a collusion attack from colluding entities.

To analyze the on-chain storage complexity, we assume the following bit-lengths as the key lengths of the cryptographic primitives to ensure 128-bit security [32]: 128-bit for the symmetric encryption algorithm, and 256-bit for the hash function and the elliptic curve pairings. The storage complexity for the parameters that must be stored in the Blockchain during the setup phase is as follows. Assuming that the total number of attributes is *U*, and each attribute is encoded in 256-bit, the storage complexity of the entire set of attributes *A* is (U× 256)-bit length. The storage complexity of the public parameters $\left(g,h_{1},h_{2},...,h_{U}\right)$ is ((U+1)× 256)-bit length, and that of the public key $PK=(e{\left(g,g\right)}^{\beta },g^{\alpha })$ is (2 × 256)-bit length. The data that need to be stored on the Blockchain after the encryption process are the metadata, including Fid and hash value H(EF), which have (2× 256)-bit length.

The computational complexity of the owner and the consumer side is as follows. Table 3 lists the notations to express the computational complexity.

The steps performed on the owner’s side are Setup, Encrypt, and KeyGen. In the Setup phase, the master secret key $MSK=g^{\beta }$ has *1 Exp* operation, and the public key $PK=({e(g,g)}^{\beta },g^{\alpha })$ has a computational complexity of *1 Pairing + 2 Exp*. When the number of owner attributes in the Encrypt phase is *l*, the symmetric encryption operation that generates EF by encrypting content *F* with the symmetric key ${sk}_{F}$ has the computational complexity of *1 Sym-enc*, and the computation of ciphertext $CT:C={sk}_{F}\cdot e{\left(g,g\right)}^{\beta s},C^{{}^{\prime }}=g^{s}$ has the complexity of *1 Pairing + (3l + 2) Exp + (2l + 2) Mul*. When the number of user attributes in the KeyGen step is *n*, the computational complexity in generating the user secret key $SK:K=g^{\beta }g^{\alpha t},L=g^{t},\forall x\in S,K_{x}=h_{x}^{t}$ is *(n + 3) Exp + 2 Mul* operations.

The steps performed on the consumer’s side are the Decrypt process. When the number of consumer attributes in this process is *m*, the computation of $\frac{e(C^{{}^{\prime }},K)}{\prod_{i\in I}({\left(e\left(C_{i},L\right)e\left(D_{i},K_{p(i)}\right)\right)}^{\omega_{i}}}$ has the complexity of *(2m + 1) Pairing + m Exp + m Mul + 1 Div*. The computation that obtains ${sk}_{F}$ has the complexity of *1 Div*. Finally, *1 Sym-dec* operation is required to recover the original data. Table 4 lists the computational complexity.

The Blockchain based DO-ABE scheme has the advantages of higher security and the utilization of hierarchical privileges in terms of data sharing; however, the computational overhead increases on the data owner’s side. However, this computational overhead does not pose a problem for the practicality of the proposed scheme. In the case of the lightweight CP-ABE scheme (number of user attributes is 600), it is reported that the encryption and decryption processes take about 3.3 ms and 6.2 ms, respectively [33]. Therefore, when applying the lightweight CP-ABE scheme such as [34,35], such amount of computational costs will not be a significant problem for practical use.

## 6. Conclusions

We presented a detailed model and process for secure and fast data sharing and transaction in vehicle networks using a Blockchain based DO-ABE. This model provides a convenient framework for data transactions between data owners and insurance companies, mobile carriers, vehicle accident investigators, and judical courts.

Blockchain-based vehicle data sharing services provide data confidentiality and integrity, privacy, and data definition as evidence for vehicle accidents, and data owners’ data control and management. This is achieved by adopting a consortium Blockchain, use of separate storages (on-chain/off-chain storage), and Blockchain based on DO-ABE with data owner-defined ACLs.

In the future, CMarket, a data marketplace platform, should be implemented through a concrete design of consensus mechanisms and implementation of smart contracts. The issue of compensation for data owners and the design of attributes and ACLs in DO-ABE remain a challenge to be studied in future research.

## Figures and Tables

**Table 1:** Data in Blocks and Transactions.

Classification	Data	Description	Size (Bytes)
Block Header	Block hash	String, SHA256	32
	Version	Integer	2
	Pre Hash	String, SHA256	32
	Tx Hash	String, SHA256	32
	nonce	Integer	4
	timestamp	Integer	4
Transaction Header	Tx ID	String	10–16
	from, to	String	41–48
	Type	String	2
	PK Hash	String, SHA1	20
	Value	Integer	8–16
	Signature	String, ECDSA using secp256r1	64
Transaction Body-Vehicle	Vehicle Metadata	Structure	maximum 256
	Blackbox Metadata	Structure	maximum 256
Transaction Body-DO-ABE	public parameter	Structure, depends on *U*	32 per each
	ACL	Arrays, depends on *U*	4 per each
	public key	String, Elliptic curve pairings	96
	Attribute Set	Arrays, depends on *U*	8 per each
	Content ID	String	10–16
	Content Hash	String, SHA256	32

**Table 2:** Comparative analysis of the existing models and the proposed model.

Category	[7]	[30]	[6]	Proposed Model
Fairness	x	Incentive Mechanism, IVTP to ensure fair payment	x	CMarket
Trasparency	Shared and Fragmented Ledger	Permissionless Blockchain	Overlay Network, Lightweight Scalable Blockchain	Permissioned Consortium Blockchain
Privacy and Secutiy	VPKI Pseudonym Certificate	IVTP’s Unique Encryption ID, Encryption Algorithm, IPFS	Changeable Public Key, Encryption Algorithm	Encryption Algorithm, Off-chain Storage
Data Owner’s Data Control and Management	x	x	Data usage can be defined	DO-ABE

**Table 3:** Computational complexity notation.

Operation	Meaning
*Sym-enc*	Symmetric key encryption such as AES
*Sym-dec*	Symmetric key decryption such as AES
*Pairing*	Bilinear pairing operation
*Exp*	Modular exponential operation
*Mul*	Modular multiplication operation
*Div*	Modular division operation

**Table 4:** Computational complexity.

Step	Computation	Complexity
Setup	$MSK=g^{\beta }$	*1 Exp*
	$PK=({e(g,g)}^{\beta },g^{\alpha })$	*1 Pairing + 2 Exp*
Encrypt(*l*)	EF= *Sym-enc* $\left(F,{sk}_{F}\right)$	*1 Sym-enc*
	$CT:C={sk}_{F}\cdot e{\left(g,g\right)}^{\beta s},C^{{}^{\prime }}=g^{s}$	*1 Pairing + (3l + 2) Exp + (2l + 2) Mul*
KeyGen(*n*)	$SK:K=g^{\beta }g^{\alpha t},L=g^{t},\forall x\in S,K_{x}=h_{x}^{t}$	*(n + 3) Exp + 2 Mul*
Decrypt(*m*)	$e(C^{{}^{\prime }},K)/\prod_{i\in I}({\left(e\left(C_{i},L\right)e\left(D_{i},K_{p(i)}\right)\right)}^{\omega i}$	*(2m+1) Pairing + m Exp + m Mul + 1 Div*
	F= *Sym-dec* $\left(EF,{sk}_{F}\right)$	*1 Sym-dec*
